# Translation regulation in response to stress

**DOI:** 10.1111/febs.17076

**Published:** 2024-02-03

**Authors:** Thomas D. Williams, Adrien Rousseau

**Affiliations:** ^1^ MRC‐PPU, School of Life Sciences University of Dundee UK; ^2^ Sir William Dunn School of Pathology University of Oxford UK

**Keywords:** mRNA, proteostasis, signalling, stress, translation

## Abstract

Cell stresses occur in a wide variety of settings: in disease, during industrial processes, and as part of normal day‐to‐day rhythms. Adaptation to these stresses requires cells to alter their proteome. Cells modify the proteins they synthesize to aid proteome adaptation. Changes in both mRNA transcription and translation contribute to altered protein synthesis. Here, we discuss the changes in translational mechanisms that occur following the onset of stress, and the impact these have on stress adaptation.

## Graphical abstract

When challenged by stress conditions such as disease, cells undergo proteome adaptation and modify the proteins they synthesize to facilitate this process. Changes in both mRNA transcription and translation contribute to altered protein synthesis. Here, we discuss the changes in translational mechanisms that occur following stress conditions, and the impact that these have on stress adaptation.

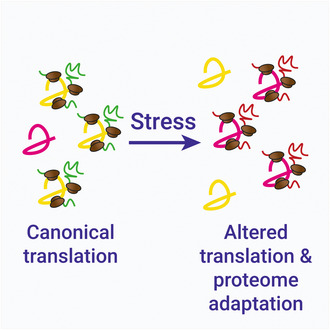

**Abbreviations**
ADP: adenosine di‐phosphate
AMP: adenosine mono‐phosphate
AMPK: AMP‐activated protein Kinase
ATP: adenosine tri‐phosphate
CDS: coding sequence
eEF: eukaryotic elongation factor
eIF: eukaryotic initiation factor
ER: endoplasmic reticulum
eRF: eukaryotic release factor
GDP: guanosine di‐phosphate
GEF: guanine nucleotide exchange factor
GTP: guanosine tri‐phosphate
HRI: haem‐regulated eIF2α KinaseGcn2: general control non‐depressible 2
IRES: internal ribosome entry site
ISR: integrated stress response
ORF: open reading frame
PABP: poly(A)‐binding protein
PB: P‐body
PERK: PKR‐like ER Kinase
PIC: pre‐initiation complex
PKA: protein kinase A
PKR: double‐stranded RNA‐dependent protein kinase
RBP: RNA‐binding protein
RiBi: ribosome biogenesis
ROS: reactive oxygen species
SG: stress granule
TC: ternary complex
TORC1: target of rapamycin complex 1, and its mammalian counterpart mTORC1
uORF: upstream open reading frame
UPR: unfolded protein response
UTR: un‐translated region

## Proteome adaptation upon stress

**Fig. 1:**
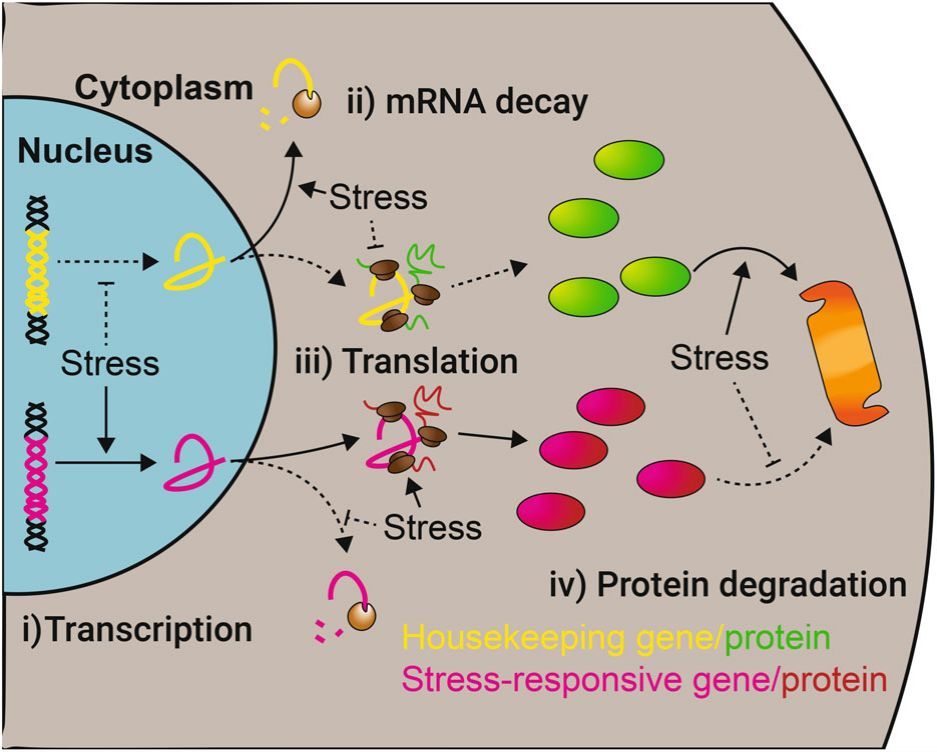
Mechanisms of proteome adaptation upon stress. (i) Transcription is altered upon stress. Production of stress‐responsive mRNAs is increased, while housekeeping mRNA production is decreased. (ii) mRNA decay further contributes to transcriptome adaptation. (iii) The altered transcriptome is subject to further regulation at the level of translation to specifically enhance production of stress‐responsive proteins. (iv) Protein degradation further aids proteome adaptation by selectively removing proteins. Dashed lines show where stresses inhibit processes. Housekeeping genes mRNAs are indicated in yellow while the proteins are in green. Stress‐responsive genes mRNAs are indicated in pink while the proteins are in red.

Environmental fluctuations cause all cells to be frequently subjected to multiple stresses in varying combinations and levels of severity. Common stresses include changes in temperature, osmolarity, reactive oxygen species and nutrient availability. To survive and proliferate, cells must adapt to and counter stresses as they arise. Altering the cellular proteome (Fig. 1) is key for stress adaptation across life [1, 2]. Extensive work has established roles for stress‐induced changes to the cellular mRNA pool across a wide variety of stresses, including in animals [3, 4, 5], plants [6, 7, 8] and fungi [9, 10, 11]. Protein production is also changed through regulation at the translational level. Alterations (or a lack thereof) in mRNA levels and translation efficiency affect protein production [12, 13]. Protein levels are further regulated by degradation, which can be induced for specific proteins upon stress [14, 15]. In this review, we discuss how mechanisms of eukaryotic translation change with stress, and the effect on mRNA translation.

## Translation in unstressed conditions

**Fig. 2:**
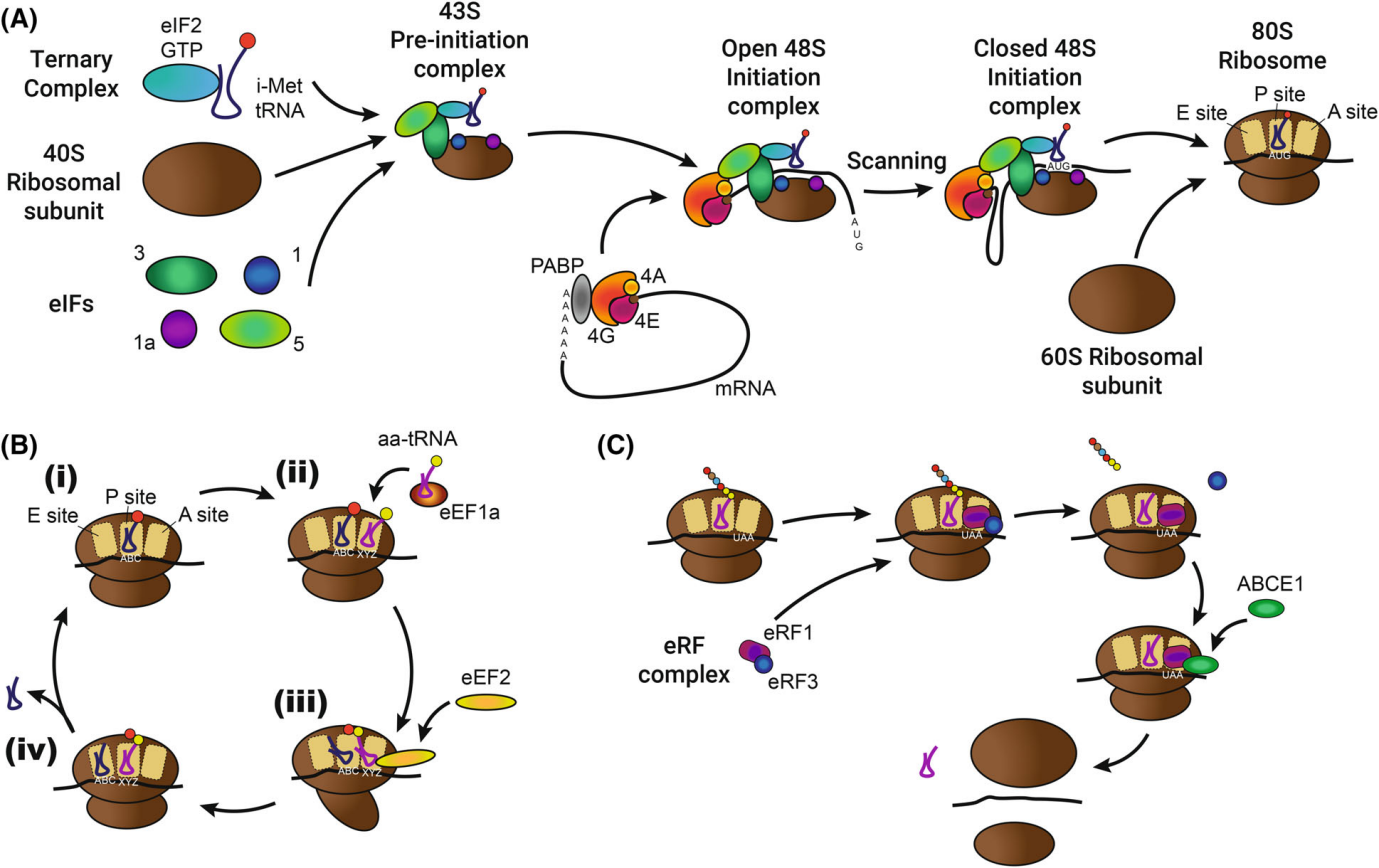
Canonical mechanism of translation. (A) Translation Initiation. The ternary complex, 40S ribosomal subunit and eIF3, eIF5, eIF1, eIF1a form the 43S pre‐initiation complex (PIC). The eIF4F complex, composed of eIF4A, eIF4G and eIF4E, binds the mRNA cap and poly‐A binding protein (PABP) to circularize the mRNA. The 43S PIC binds the eIF4F complex and scans along the mRNA until the initiator methionine (i‐met) tRNA of the ternary complex recognizes an AUG codon. The 60S ribosomal subunit is then recruited to form the 80S ribosome with the i‐met tRNA in the peptidyl transferase (P)‐site. (B) Translation elongation. (i) Following initiation or a round of translation, a ribosome has one tRNA in the P‐site. (ii) A codon‐matched amino‐acylated (aa‐)tRNA is recruited to the acceptor (A)‐site by eEF1a. (iii–iv) mRNA translocation occurs, mediated by eEF2, moving the tRNAs from the P‐ to the Exit (E)‐site, and A‐ to P‐site, adding one amino acid to the peptide chain. The E‐site tRNA is released and the process repeats from (i) until a stop codon is reached. (C) Translation termination. Once a stop codon is reached, it is recognized by the eRF1/3 complex, which causes the peptide chain to be released. The ribosome is then split for recycling.

Canonical translation (Fig. 2) is widely described, with initiation considered the major rate‐limiting step [16, 17]. To initiate translation (Fig. 2A), a ternary complex (TC) consisting of initiator‐methionine (i‐met) tRNA, eIF2 and GTP is formed. This binds to the 40S small ribosomal subunit through eIF3 and eIF5. eIF1 and eIF1A also bind to the 40S to form the 43S pre‐initiation complex (PIC). The PIC binds the eIF4F cap‐binding complex (composed of eIF4G, eIF4E and eIF4A) on the 5′cap of an available mRNA to form the 48S initiation complex. This interaction is mediated through eIF3 and eIF5 binding to eIF4G, while ATP‐bound eIF4A allows formation of an open complex [18, 19, 20]. The 48S complex initiates scanning along the mRNA in this open conformation, with the eIF4A helicase unwinding the mRNA secondary structures, facilitated by its interaction with eIF4B [21]. Whether the eIF4F complex remains bound to the cap (or alternatively if eIF4E disconnects from the other eIF4F components while remaining bound to the cap) during scanning is an open question. Notable, but relatively small, 40S mRNA footprints indicative of queueing occurring during scanning are observed, suggesting that this disconnection from the cap can occur, allowing multiple PIC complexes in the mRNA 5′ UTR [22, 23]. Binding of eIF4E to the PIC increases scanning activity of the eIF4A helicase, implying a benefit for maintenance of the connection between the complete eIF4F complex and PIC [24]. The exact scanning mechanism may vary according to factors including mRNA secondary structure and modifications, although this remains to be determined. Once a suitable start codon is identified via codon‐matching with the i‐met tRNA, the TC's GTP is hydrolysed to GDP, and eIF2 is released. The 48S shifts from an open to a closed conformation, locking i‐met tRNA and mRNA together at the peptidyl transferase (P‐) site [25]. Initiation factors are replaced by the 60S large ribosomal subunit to form the 80S ribosome via an intermediate complex containing eIF5B [26].

During translation elongation (Fig. 2B), codon‐matched charged tRNAs, delivered by eEF1A, bind the mRNA in the aminoacyl (A‐) site of the ribosome [27]. mRNA translocation occurs via 40S conformational changes, assisted by eEF2, which move the tRNA from the A‐site to the P‐site [28, 29]. The tRNA‐conjugated amino acid is added to the C‐terminal end of the peptide chain and translocation occurs again to move the free tRNA to the exit (E‐) site, where it is ejected. A new tRNA can then bind in the A‐site to restart this process.

When a stop codon is reached, there are no codon‐matched tRNAs, so translation termination and ribosome disassembly occur (Fig. 2C). The eukaryotic release factor complex (composed of eRF1 and eRF3‐GTP) binds in the A‐site and terminates protein synthesis by hydrolysing the bond between the final tRNA and its amino acid [30, 31, 32]. Subsequent release of eRF3 and binding of an ABC‐ATPase protein and ATP hydrolysis splits the 80S back into 40S and 60S subunits, which are recycled for subsequent rounds of translation [33, 34]. Poly(A)‐binding protein (PABP) binds eIF4G, eIF4B and the 3′ mRNA poly(A) tail to: circularize the mRNA; stabilize the interaction of eIF4E with the 5′cap and boost translation termination efficiency to boost ribosome occupancy and translation [35, 36, 37, 38]. Closed loop formation is particularly biased towards shorter mRNAs [39]. Further, PABP can interact with both 40S and 60S ribosomal subunits, potentially limiting their diffusion away from the mRNA and promoting re‐initiation [40].

**Fig. 3:**
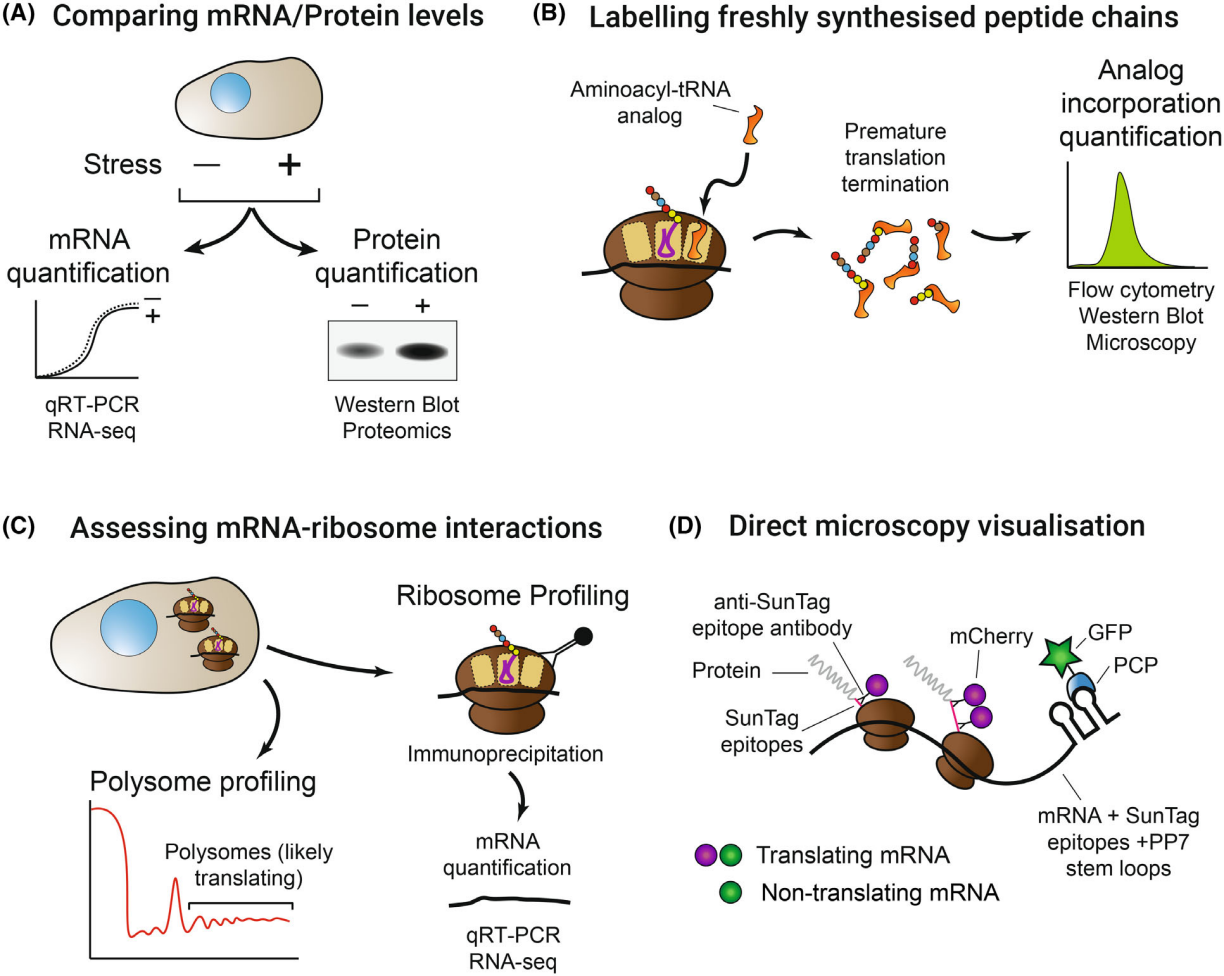
Methods to measure translation. (A) Comparing mRNA and protein levels to look for changes in translation of either one specific protein or multiple proteins using genomic and proteomic techniques. Protein degradation must be accounted for. (B) Labelling freshly synthesized peptide chains with aminoacyl‐tRNA analogues or labelled amino acids and quantifying the incorporation. (C) Assessing mRNA–ribosome interactions, either of specific mRNAs or transcriptome wide. (D) Direct microscopy visualization of translation of specific mRNAs through technologies such as SunTag, shown, which uses colocalization analysis of nascent chain (labelled with a fluorescent anti‐SunTag nanobody) and mRNA (labelled with green‐fluorescent protein tagged PP7 coat protein, PCP‐GFP).

Many of these mechanisms are altered in either efficiency or character upon stress to change the rates and selection of mRNA for translation. Below, we discuss how changes in mRNA availability and mechanisms of translation affect the pool of translating mRNAs and protein production. In Box 1 and Fig. 3, we highlight some techniques and approaches which can be used to assess translation so researchers new to the field can get an overview of available methodologies.

Box 1 **Techniques to monitor translation**

Translation is a highly dynamic, multi‐step process, readouts of which can be impacted by other processes (e.g. protein degradation). A further complicating factor is that there is no fixed rate of translation: much like transcription, translation occurs in bursts with periods of high and low activity [41, 42]. A large variety of techniques can be employed to measure translation; from the whole translatome to individual mRNAs. These range from comparing protein and mRNA levels to determine how much protein is produced per mRNA, while recognizing and controlling for the contribution of protein degradation (Fig. 3A) [43], incorporation of radioactive amino acids, chemically modified amino acids, or aminoacyl tRNA analogues into newly synthesized peptide chains (Fig. 3B) [44, 45, 46, 47, 48], investigation of mRNA–ribosome interactions (Fig. 3C) [49, 50, 51], and live or fixed cell microscopy of specific mRNA translation (Fig. 3D) [41, 52, 53]. Each technique has advantages and disadvantages for determining mRNA translation efficiency, and each requires a healthy degree of scepticism and appropriate controls when interpreting results due to several additional factors which can impact the observations. Researchers wishing to explore translation for the first time are advised to look through the methods highlighted here to help identify the one(s) most appropriate for their particular question.

## Effect of stresses on mRNA availability

### Stress‐induced formation of RNA‐containing granules

For translation to occur, both the translation machinery and mRNA must be available. Several stresses cause rapid sequestration of these components into stress granules (SGs: mRNA, translation initiation complexes and RNA binding proteins (RBPs)) and P‐bodies (PBs: mRNA and RBPs such as those involved in mRNA decay) mediated by liquid–liquid phase separation of RBPs [54, 55]. While SGs formed upon different stresses share a name and common components, there are significant compositional differences. SGs formed following eIF4A inhibition have relatively low levels of mRNAs, eIF4G and eIF3B compared to SGs formed following sodium arsenite treatment [56]. The importance of stress‐induced eIF2α phosphorylation to SG formation is also context dependent [56]. Care must, therefore, be taken when extrapolating SG function between stresses. Although most mRNAs in SGs are translationally repressed, stress‐activated (*ATF4*) and inhibited (5′TOP containing *RPL32*) mRNAs can be translated within these structures, with only a moderate decrease in the translationally active proportion compared to cytosolic mRNAs [57]. Phase‐separated granules supporting translation in non‐stressful conditions have also been widely reported across eukaryotes [58, 59, 60, 61, 62, 63]. Microscopy resolution remains a limiting factor for identifying small and/or more diffuse stress granules over the cytosolic background. While fundamental questions are being re‐opened about the nature of SGs as a translationally inactive mRNA storage compartment, further investigations are required to determine whether, and to what extent, other mRNAs are translated within SGs.

In contrast to SGs, PBs are devoid of translating mRNA and translation factors, instead containing proteins associated with mRNA decay and translational repression [57, 64]. Despite this, PBs do not seem to be sites of general mRNA degradation, and mRNA decay occurs when PB formation is prevented [65, 66]. Indeed, some mRNAs are stabilized upon stresses which induce PB formation: possibly by PBs sequestering mRNA degrading enzymes [66, 67, 68]. This has larger effects on some mRNAs (e.g. nonsense‐mediated decay‐regulated mRNAs) than others. Degradation of other mRNAs is regulated by alternative splicing, notably the *HAC1* mRNA in *S. cerevisiae* (*XBP1* in mammals) following ER stress, alternative splicing of which prevents degradation, allowing translation and unfolded protein response (UPR) initiation [69, 70]. Other mRNAs are similarly regulated [71, 72, 73]. Together with alterations at the transcriptional level, altered mRNA decay and splicing change the pool of mRNAs available for translation. For some mRNAs, this may be important to offset changes to translation efficiency, while for others it is the primary way of regulating protein output [74].

### Stress regulation of mRNA–RNA‐binding protein interactions

mRNAs interact extensively with RNA‐binding proteins (RBPs), which can regulate mRNA translational capacity [75]. This includes, but is not limited to, factors involved in translation initiation, many of which have reduced mRNA association following stress [76, 77]. Other proteins known to affect translation, such as the *S. cerevisiae* translational repressor Puf3, have altered overall levels bound to mRNA following stress [76]. In most cases, additional studies to determine the specific mRNAs these proteins differentially interact with are yet to be performed. In follow‐up studies which have been performed, work has focused on either identifying mRNAs bound to a particular protein or using genetic manipulation followed by assessment of protein levels [78, 79]. Other stress‐regulated RBPs include metabolic proteins, such as thymidylate synthase and iron regulatory proteins, some of which regulate levels of certain proteins, including their own, either through regulation of mRNA stability or translation initiation [80, 81]. While many RBPs contain defined RNA recognition motifs, many do not. It is becoming more apparent that proteins without canonical RNA recognition motifs can interact with diverse mRNAs through phase separation, either in SGs/PBs or other similar bodies, and are thus able to manipulate their translation and sequestration into these structures [63, 82, 83, 84]. The molecular properties of the mRNAs which facilitate this remain unclear, although mRNA is more effective than other types of RNA at interacting with phase‐separated bodies, perhaps indicating an importance for both length and a relative lack of secondary structure [84].

Additional RNA‐containing structures involved in translation can have altered interactions with proteins which affect their function. This includes different ribosomal protein paralogues (discussed in the ‘translation elongation’ section and reviewed in [85]), and tRNAs. To deliver amino acids to the ribosome, tRNAs require aminoacylating. Under stress conditions, methionine‐tRNA‐synthetase loses specificity and mis‐charges non‐Met tRNAs with methionine, which can be used in translation. This occurs across species, possibly to aid in countering increased ROS [86, 87, 88]. Under certain stress conditions, tRNAs can be bound and cleaved by angiogenin, removing them from the tRNA pool [89]. Altered interactions between RNAs, tRNA and RBPs following stress have major impacts on translation.

## Effect of stresses on translation initiation

**Fig. 4:**
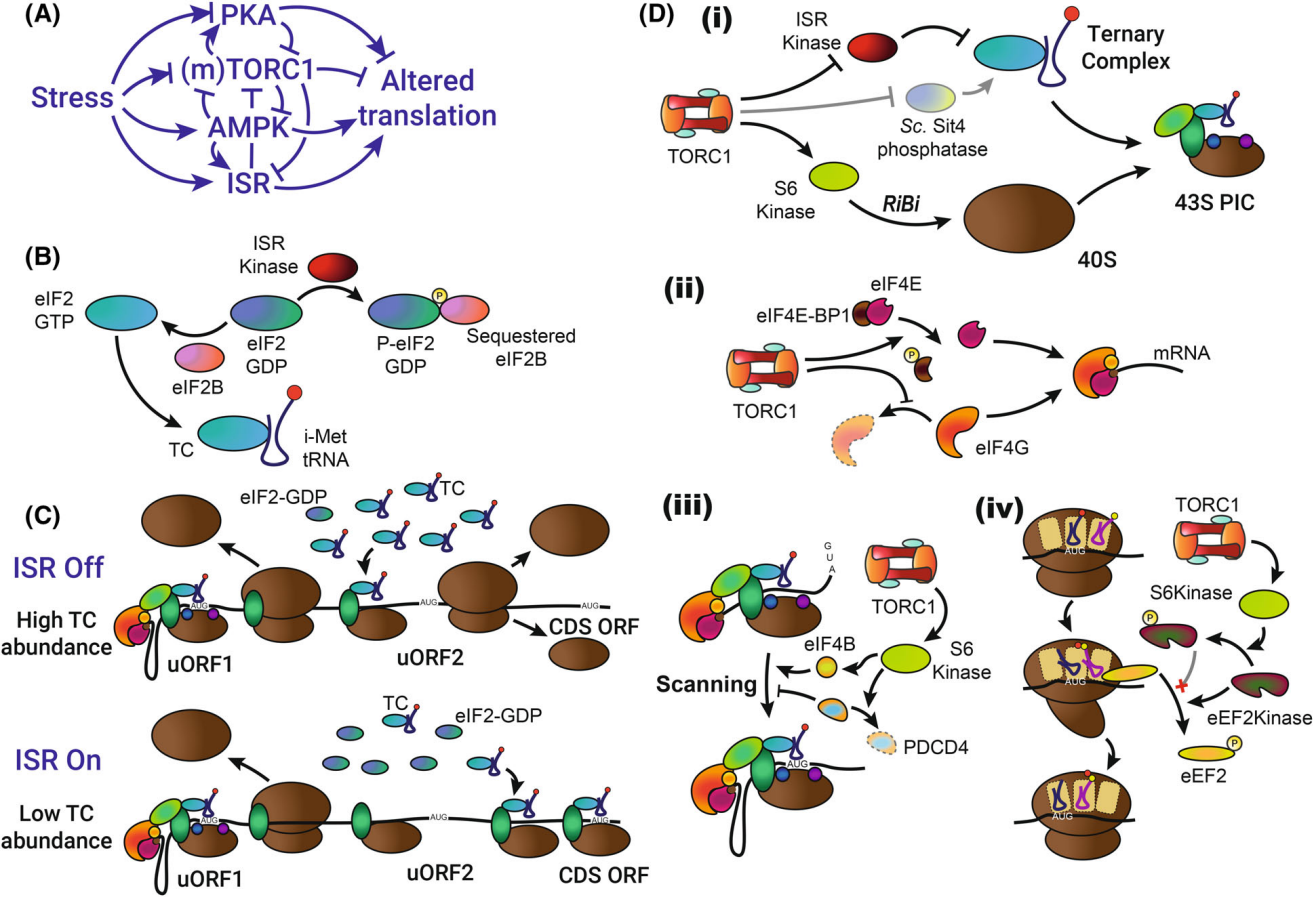
Changes to translation upon stress. (A) Stress impacts signalling from various kinases to alter translation. Arrowheads indicate activation, while barred lines indicate inhibition. ISR is the integrated stress response. (B) Inhibition of ternary complex formation by the ISR. ISR kinases phosphorylate eIF2α to sequester the eIF2 GEF and eIF2B, preventing GDP to GTP transition and re‐binding of initiator tRNA. (C) Regulation of *ATF4/GCN4* translation by upstream open reading frames (ORFs) is dependent on ISR signalling. Following initiation and termination from uORF1, the restriction of ternary complex (TC) availability under conditions of ISR activation prevents premature re‐initiation at uORF2. This allows translation initiation from the start codon of the coding sequence (CDS) ORF. In mouse *ATF4* mRNA, uORF2 overlaps the CDS ORF. For *GCN4* mRNA, the uORF1 represented here is analogous to the first ORF pair (uORFs1&2) while uORF2 is analogous to the second ORF pair (uORFs3&4). (D) Impacts of the stress‐inhibited kinase TORC1 signalling on translation. (i) TORC1 inhibition restricts 43S pre‐initiation complex formation through ISR activation (see Fig. 4B) and reduced ribosome biogenesis (RiBi) through decreased S6Kinase activation. In *S. cerevisiae*, the TORC1‐inhibited phosphatase Sit4 can counter the ISR kinases. (ii) TORC1 inhibition restricts availability of the eIF4F components eIF4G (via increased degradation) and eIF4E (through eIF4E‐BP1 sequestration). (iii) TORC1 inhibition, and reduced S6Kinase activation, decreases eIF4B activation of eIF4A and prevents degradation of the eIF4A inhibitor PDCD4. (iv) Inhibition of TORC1, and resulting S6Kinase inhibition, allows eEF2Kinase to inhibit the ability of eEF2 in mRNA translocation.

Translation initiation is regulated by altered cellular signalling upon stress: principally through changing translation initiation component availability. The major stress‐regulated signalling pathways involved in altered translation upon stress are highly evolutionarily conserved. These include the integrated stress response (ISR) kinases, TORC1 (target of rapamycin complex 1 and its mammalian counterpart mTORC1 – collectively referred to here as TORC1), AMP‐activated protein kinase (AMPK) and protein kinase A (PKA). The regulation of these kinases is multi‐layered with substantial interplay (Fig. 4A). While we discuss the roles of these kinases individually, their interplay is key to producing complex and highly regulated changes to cellular translation. For example, both TORC1 inhibition and ISR activation are necessary for the expression of Nanog‐291 and Snail‐85 mRNAs upon stress simulation in breast cancer cells [90]. Depending upon the mRNA and its specific properties, translation can either be up‐ or downregulated by the same altered signalling, allowing a consolidated translational response to the stress. This response can change over the lifetime of the stress with different signalling outputs apparent in cells under acute and chronic stress [91, 92, 93, 94]. In the following sections, we detail these and other changes to the mechanisms and location of translation initiation.

### Integrated stress response

The ISR is mediated by kinases which reduce, but do not eliminate, ternary complex (TC) assembly through inhibiting the ability of eIF2 to bind GTP [95]. The mechanisms of ISR activation are varied. In mammalian cells, the ISR is triggered by the activation of any one of four kinases (PERK, PKR, HRI, Gcn2) which are descended from the ancestral Gcn2, the only form found in yeast [96, 97]. PERK is activated by ER stress and hypoxia [98, 99, 100]. PKR is activated by double‐stranded RNA and interferon signalling (notably during viral infection), and possibly ER and oxidative stresses, although the evidence is circumstantial [101, 102, 103, 104]. HRI is activated by heat, osmotic, oxidative, haem deficiency, mitochondrial and proteotoxic stresses [99, 105, 106, 107, 108, 109, 110, 111]. Gcn2 is regulated by amino acid availability, interaction with the P‐stalk of non‐translating ribosomes, cytoskeletal perturbation, infection, TORC1 regulation of downstream phosphatases and phosphorylation by AMPK, in addition to oxidative, and possibly other stresses, in yeasts [112, 113, 114, 115, 116, 117, 118, 119, 120]. In the most well‐described mechanism of Gcn2 activation, Gcn2 interacts with Gcn1 and the yeast‐specific Gcn20, which recruit free tRNAs to Gcn2 at the ribosomal A‐site: a reaction that is enhanced under conditions where the availability of an alternative binding partner is limited (Yin1/IMPACT for Gcn1, eEF1α for Gcn2) [119, 121, 122, 123]. Gcn2 has additionally been described as a TORC1 inhibitor through regulating translation of Sestrin2 (which interferes with TORC1 localization), eIF2α phosphorylation and potentially by directly phosphorylating TORC1 itself [124, 125, 126].

Activated ISR kinases phosphorylate the eIF2 complex subunit eIF2α at Serine 51. This phosphorylation creates an alternate binding site for the eIF2 guanine nucleotide exchange factor (GEF) eIF2B, sequestering it and preventing GDP to GTP exchange on eIF2 [127]. GDP‐bound eIF2 has much lower affinity for the i‐Met tRNA and thus TC formation is inhibited (Fig. 4B) [128]. Reduced TC availability rapidly decreases translation initiation, allowing the cell time for transcriptome modification for stress‐adaptive translation. It additionally acts to decrease production of proteins which are liable to exacerbate stresses, such as the mitochondrial complex I protein NDUFAF2: the decrease of which limits oxidative stress [129]. Activation of the ISR during parasite infection can lead to reduced host‐cell nutrient usage, thereby enhancing pathogen growth, while for other infections the ISR inhibits pathogen multiplication [120, 130].

While translation initiation is decreased by eIF2α phosphorylation, TC levels are reduced rather than absent, allowing some initiation to occur. By modulating the level of ISR activation, cells can fine‐tune TC availability as appropriate for the conditions [131]. Altered TC availability does not uniformly reduce translation, and thereby changes the proteome composition in cells with activated ISR. Altered translation in response to ISR activation is best described for the upstream ORF (uORF)‐regulated transcription factors ATF4 and its yeast equivalent Gcn4. In mouse *ATF4* mRNA, there are two uORFs: one distal from the start codon, and one proximal which encodes a peptide overlapping the start codon (human *ATF4* mRNA contains three uORFs: two distal and one proximal). *GCN4* has four uORFs, which can be thought of as occurring in pairs (1 and 2, 3 and 4) with similar functionality to those in *ATF4* mRNA [97].

*GCN4*/*ATF4* mRNA translation (Fig. 4C) initiates at the distal uORF(s) through canonical translation mechanisms and swiftly terminates. Following translation termination, a subset of the 40S ribosomes remains bound to the mRNA, aided by an interaction between a sequence‐specific part of the mRNA and eIF3, which can remain bound to the 40S for the initial ~ 75 amino acids [132, 133]. These 40S complexes resume scanning and reacquire a TC, which can interact with eIF3, to initiate at a downstream site [134]. When the TC is more abundant, this occurs rapidly, and translation is initiated at the proximal uORFs. Following termination at these uORFs, the 40S does not maintain its mRNA interaction and no further reinitiation occurs. When the TC is less abundant, it takes longer to acquire a new TC. Scanning consequently proceeds past the uORFs and initiates at the *GCN4/ATF4* start codon. Spacing between the uORFs is critical for this regulation [135]. Delivery of i‐Met tRNA can be performed upon TC formation inhibition by the eIF2D/DENR complex, in the absence of which *ATF4* expression is drastically reduced in *Drosophila* fat cells [136]. In contrast, eIF2D recycles 40S subunits and inhibits reinitiation in *S. cerevisiae*, although this role may be sequence dependent [137]. It is important to acknowledge that not all uORFs are involved in translational regulation: recent evidence indicates that only a small, albeit important, subset of uORFs regulate translation [138]. Other mRNAs with only one inhibitory uORF are more likely to have this uORF bypassed under stress [139]. Specific translation of Gcn4/ATF4 and other transcription factors allows transcriptional rewiring prior to the resumption of translation from a newly stress adapted transcriptome, contributing to stress adaptation.

Intriguingly, despite the TC's importance for translation initiation, inducing eIF2α phosphorylation in the absence of stress does not necessarily inhibit translation [140, 141]. Analysis of a mutant which sustains bulk translation at normal rates upon TORC1 inhibition despite high levels of phosphorylated eIF2α implicated defective regulation of eIF4G, which has been recently linked to isoform‐specific stress adaptive translation [141, 142]. Furthermore, other studies have shown that upon prolonged stress ATF4 expression can decrease while eIF2α phosphorylation remains high, potentially due to a reduction in the number of ribosomes [91, 92]. Underscoring the interplay between the different signalling elements discussed here, ATF4 protein expression can also be induced by activating TORC1 in a poorly understood process dependent on eIF4E availability and involving mRNA stabilization and increased uORF skipping independently of TC availability, although the transcriptional targets are distinct [143, 144]. Countering the ISR are the PP1 phosphatases which are active against P‐eIF2α when associated with either the constitutively expressed CReP (also known as PPP1R15B) regulatory subunit, or the ISR‐induced GADD34 (also known as PPP1R15A) regulatory subunit and G‐actin, which helps stabilize the complex [145, 146, 147]. In this way, the ISR acts to limit its own activation. Therefore, while great importance is ascribed to ISR activation, it is important to remember that this always occurs within a network of multiple interacting regulators (Fig. 4A).

### Target of rapamycin complex 1

TORC1 regulates various downstream signalling pathways, or branches, which collectively promote anabolic, and inhibit catabolic, processes when TORC1 is active [148]. These branches include autophagy, S6Kinases and PP2A phosphatases, regulation of which are highly conserved from yeast to humans, demonstrating a central role in eukaryotic biology.

TORC1 signalling couples environmental status and nutrient availability with anabolic functions essential for cell growth, including translation. TORC1 activity towards either of the two most investigated downstream effectors, S6Kinase and eIF4E‐BP1, is inhibited by various stresses, including oxidative, envelope, hypoxic, osmotic, nutrient, heat, metabolic and ER stresses [9, 12, 149, 150, 151, 152, 153, 154, 155, 156, 157]. S6Kinase signalling can also be activated by mild oxidative and ER stresses [149, 158]. In mild stress conditions of nutrient limitation, akin to those found in the wild, *S. cerevisiae* TORC1 activity becomes oscillatory [93]. Other TORC1 effectors are regulated by some, but not all, of these stresses [9]. TORC1 activity is, thus, highly responsive to changing cellular conditions.

The majority of TORC1 is activated in the presence of amino acids through recruitment to the lysosome/vacuole by a GTPase complex. Once there, TORC1 is activated by the Rheb small GTPase, which is responsive to environmental status, particularly the presence of growth factors in mammalian cells. The link between Rheb and TORC1 has been lost in *S. cerevisiae* (although not *S. pombe*) and various other lower organisms, indicating that environmental status can be sensed through alternative mechanisms such as AMPK activation [159, 160, 161]. No Rheb homologue in plants is known, although a putative RhebGEF with the expected distribution and effect on growth has been identified [162]. While most TORC1 is at the lysosome/vacuole, a minority is found elsewhere in the cell, with these pools likely to have their own distinct functional effects and downstream signalling [163, 164, 165]. Little is known about how these non‐lysosomal/vacuolar pools are regulated and respond to stress, although Rheb localized to alternative membranes and the nucleoplasm is likely involved [165, 166, 167].

TORC1 inhibition restricts subunit availability for the PIC and eIF4F complexes, as well as scanning and elongation (Fig. 4D). TORC1 inhibition activates the ISR by Gcn2 dephosphorylation through the PP6C phosphatase in mammalian cells, and an unidentified phosphatase in *S. cerevisiae* [114, 115]. ISR activation, in turn, can inhibit TORC1 [124, 125, 126]. The *S. cerevisiae* PP2A phosphatase Sit4, which is activated by TORC1 inhibition, can counter ISR kinases to increase TC availability [113]. Further restriction of PIC formation (Fig. 4Di) is mediated by the S6Kinase branch of TORC1 signalling controlling the ribosome biogenesis (RiBi) transcriptional programme [168]. S6Kinases are named for their ability to phosphorylate ribosomal protein S6 (Rps6). In mammalian cells, Rps6 phosphorylation itself, likely in the nucleus, is important for RiBi, although in *S. cerevisiae*, this regulation occurs primarily through the S6Kinase Sch9 regulating transcriptional repressors, with the alternate S6Kinase Ypk3 being responsible for the majority of Rps6 phosphorylation [169, 170, 171, 172, 173]. Phosphorylated Rps6 in the 40S may aid in translation initiation, with a potential bias for shorter and 5′TOP mRNAs [174].

Availability of the eIF4F cap‐binding complex, composed of eIF4A, eIF4E and eIF4G, is regulated by TORC1 signalling (Fig. 4Dii). eIF4E, the cap‐binding subunit, is alternately bound by eIF4E‐binding proteins, most notably eIF4E‐BP1 in mammalian cells. TORC1 directly phosphorylates eIF4E‐BP1, preventing this binding from occurring and allowing eIF4F complex formation [175]. When TORC1 activity is reduced, eIF4E‐BP1 phosphorylation is reduced, allowing it to bind and sequester eIF4E, limiting translation initiation. Intriguingly, unlike the majority of TORC1 signalling events, the evolutionary history of eIF4E binding protein phosphorylation remains unclear. eIF4E binding proteins in yeasts and nematodes are not thought to be regulated by TORC1, unlike in flies, mammalian cells and amoebae [176, 177, 178, 179]. However, a recent report found that the *S. cerevisiae* phosphorylation of the eIF4E‐binding protein Caf20 is regulated by TORC1, although this does not cause eIF4E sequestration [180]. TORC1 activity controls the phosphorylation state of the eIF4F member eIF4G, which helps recruit the PIC to mRNA [44, 181]. *S. cerevisiae* eIF4G levels are controlled by TORC1 through autophagy‐mediated degradation, while mammalian eIF4G is degraded in a caspase‐dependent manner in conditions where TORC1 is inhibited, although whether the caspases act on eIF4G directly, or through promotion of autophagy remains unknown [141, 182, 183]. Intriguingly, the eIF4F complex also physically interacts with TORC1 and can facilitate reduced TORC1 activity in amino‐acid‐starved conditions [184, 185].

TORC1 promotes scanning and elongation via S6Kinase activation (Fig. 4Diii–iv). S6Kinase enhances phosphorylation of eIF4B, which then promotes eIF4A helicase activity and translational scanning [186]. S6Kinase further promotes degradation of the eIF4A inhibitor PDCD4 [187]. eEF2Kinase is phosphorylated and inactivated by S6Kinase, preventing it from inhibiting eEF2 [188]. Although this would be expected to mostly impact elongation, eEF2 activity is also important for ribosome recycling and translation initiation [189]. S6Kinase inhibition is equally important for decreasing translation in stressed cells: a phospho‐mimetic form of the *S. cerevisiae* S6Kinase Sch9 (simulating the ‘TORC1 active’ state) largely prevents translation downregulation, assessed by polysome profiling, upon TORC1 inhibition [171]. While TORC1 activity has a major role in regulating eIF4F‐dependent translation initiation, the effect of TORC1 inhibition, and its signalling branches, on translation in response to most stresses remains to be determined.

### AMP‐activated protein kinase

AMPK is activated in response to stresses which reduce cellular ability to produce ATP (including hypoxia, amino acid starvation and TORC1 inhibition) by binding AMP/ADP [190, 191]. AMPK activation affects translation by both inhibiting TORC1, its downstream S6Kinase, and activating the ISR [113, 155, 156, 159]. AMPK activation by exercise is associated with eIF4E‐BP1 dephosphorylation, causing a change in the translation of certain mRNAs, although it remains unproven whether this is AMPK dependent and whether the pathway is mediated through TORC1 activity modulation or a parallel mechanism [192]. AMPK further promotes eEF2 phosphorylation, inhibiting translation elongation and ribosome recycling, by both activating eEF2Kinase and sequestering the eEF2Kinase phosphatase PP6C, which is intriguingly activated by TORC1 inhibition [114, 189, 193, 194].

### Protein kinase A

PKA is regulated by intracellular levels of cAMP, becoming activated when cAMP binds the inhibitory regulatory subunits, releasing the catalytic subunits [195, 196]. Glucose stimulates cAMP production by boosting ATP generation, leading to PKA activation in *S. cerevisiae* [196, 197, 198]. While glucose induces mammalian PKA activity towards some substrates, glucose withdrawal can also stimulate PKA [199, 200, 201]. Glucose withdrawal causes both autophagic degradation of a PKA inhibitory subunit and ER stress, another PKA‐activating signal [200, 202, 203]. PKA interacts with other stress‐regulated signalling: TORC1 relieves PKA inhibition in *S. cerevisiae*, while in mammalian cells, PKA inhibits TORC1 and AMPK signalling [199, 204, 205, 206]. TORC1 and PKA have substantially overlapping downstream target proteins outlining a similar function in promoting cellular anabolism [207]. PKA phosphorylates eIF4B, likely promoting eIF4A activity and thus translation initiation [208]. Counterintuitively, the *S. cerevisiae* PKA catalytic subunits Tpk2 and Tpk3 are required for translation inhibition upon glucose starvation, and affect translation of certain mRNAs following heat stress: possibly by recruiting specific eIF4G subunits, which can affect stress‐adaptive translation, to the eIF4F complex [142, 209, 210, 211]. The PKA response to stress is multi‐faceted, with a deeper knowledge of the subsequent impacts upon translation still a work in progress. The multitude of downstream targets makes this work exceedingly challenging.

### eIF4F‐independent translation initiation

Although ribosome recruitment via the eIF4F complex is the dominant mode of ribosome docking onto mRNA, alternatives exist. These alternatives become particularly relevant following stress when many components of the eIF4F complex are depleted either through degradation (eIF4G), sequestration (eIF4E) or dissociation (eIF4A) [141, 175, 212, 213].

Following viral infection cells shut down translation initiation to try to restrict viral amplification. To circumvent this, many viral mRNAs contain internal ribosome entry sites (IRESs), mRNA secondary structures which can recruit ribosomes in the absence of eIF4F bound to an mRNA 5′ cap [17, 214]. In at least some instances, IRES binding of eIF3 is involved in this recruitment, while other IRES sequences can recruit eIF4F components [215]. Interestingly, an IRES does not have to be upstream of the start AUG codon to promote translation: ribosome recruitment to the 3′UTR can drive increased translation by facilitating ribosome delivery to the 5′UTR [216]. There are additionally hundreds of known eukaryotic IRESs, although care must be taken before concluding that any sequence has IRES activity [217, 218].

While IRESs frequently contain large structural elements, they do not have to be structured. PABPs, which bind to both eIF4G and A‐rich sequences, can bind to unstructured A‐rich 5′UTRs in mRNAs and recruit ribosomes for increased translation upon stress [219, 220]. As different eIF4G proteins are differentially involved in stress‐adaptive translation from mammalian to yeast cells, it is possible that isoform‐specific differences in the PABP binding region could regulate IRES translation of these targets [142]. A‐rich mRNA sequences can also be methylated upon stress by METTL3, allowing binding of ABCF1 to the mRNA in the 5′UTR away from the cap. ABCF1 likely recruits eIF4G for cap‐independent initiation [221]. IRES sequences can circumvent eIF4F‐dependent translation initiation downregulation (thus maintaining translation) or upregulate translation of IRES‐containing mRNAs following stress [222]. IRESs can even facilitate translation of different protein isoforms [223].

eIF3, in addition to the ability to promote IRES‐regulated translation, can bind the mRNA cap and recruit ribosomes for eIF3‐dependent translation through the eIF3d subunit [224]. This method of ribosome recruitment becomes a major route for translation initiation for cells under various stresses [225, 226, 227]. Whether there is a bias of eIF3d binding towards stress‐adaptive mRNAs remains unknown. Recruitment of ribosomes to mRNA in such a distinct manner is likely to contribute to different translational efficiency of mRNAs following stress.

### Stress‐induced translation from alternative start codons

While we have so far discussed translation initiation at AUG sites, initiation can also occur at alternative start codons, particularly at similar sequences like CUG [228]. Translation initiation site mapping indicates that up to 50% of translation initiation occurs at non‐AUG sites. Although it is possible that many of these sites are either initiation errors or artefacts of the experimental conditions, many likely encode short ORFs with regulatory functions [229]. A recent excellent review on this topic is available elsewhere [230]. Translation from non‐AUG start codons can be enhanced upon stress, including from two examples upstream of uORF1 on *GCN4* mRNA, which may help promote Gcn4 expression [231]. In mammalian cells, the mitochondrial ribosome protein L18 can be translated from a downstream CUG codon upon stress to generate a cytosolic isoform, which helps promote heat shock protein translation [232]. Translation from non‐AUG initiation sites, therefore, may facilitate translational adaptation to stress.

### Translation of stress‐adaptive mRNAs at translation hot spots

To boost translation upon stress, defined mRNAs can be recruited to translational platforms, including at regions of dense actin [1, 63, 82, 233, 234, 235]. Several proteins involved with these platforms have a high propensity for phase separation. Stress conditions which promote mRNA relocalization to SGs/PBs may similarly promote localization of certain mRNAs to these regions to increase stress‐responsive translation initiation. Similar phase‐separation‐driven promotion of the translation of translation factors is observed in unstressed conditions [59]. Such an increase in translation could be mediated by local activation of mTORC1 allowing increased local availability of the eIF4E cap‐binding protein for longer bursts of active translation, although this remains speculative [41, 236]. Evidence of whether these hotspots occur in mammalian cells under stress, and how extensively they are used in *S. cerevisiae* remains to be established.

## Effect of stresses on translation elongation

For efficient elongation, there must be sufficient codon‐matched aminoacylated tRNAs alongside space in the A‐site for them to bind. eEF2 is important for translocation of the ribosome, thus vacating the A‐site for the subsequent round of tRNA binding. eEF2 activity is inhibited by phosphorylation from eEF2Kinase which has low activity under unstressed conditions. eEF2Kinase activity is constrained by phosphorylation from the TORC1‐activated S6Kinase (Fig. 4Div) [188, 237]. Upon stress, eEF2Kinase is no longer repressed but is activated by AMPK [194]. eEF2Kinase then phosphorylates and inhibits eEF2. This inhibition is countered by the TORC1‐repressed phosphatase PP6C, which activated AMPK sequesters [114, 193]. This interplay allows fine‐tuning of eEF2 activity according to the type and severity of stress.

tRNAs can be modified under stress conditions to allow for greater wobble at the third base, mitigating against reduced tRNA availability and promoting translation of select transcripts [238, 239]. For some tRNAs, this is insufficient: upon oxidative stress there is a reduction in Trp tRNA availability, leading to ribosome stalling and collisions at the single Trp UGG codon [240]. The increase in uncharged Trp tRNAs additionally leads to ISR activation, affecting translation initiation. Increased collisions could lead to erroneous translation through frameshifting, but this is mitigated by ribosome‐bound factors such as Slf1 [241].

Several ribosomal proteins have paralogues, resulting in numerous possible ‘flavours’ of ribosome, which have different preferences for mRNAs to translate [85]. Such a role has been reported in different tissues, and within single cells [242, 243]. Modulation of ribosome heterogeneity can be controlled by post‐translational modifications affecting ribosomal proteins or rRNA, with impacts upon mRNA translation [244, 245, 246]. This can be at the level of elongation rates through codon selection or through initial mRNA binding, including through IRESs [243, 246, 247]. Switching of the prevalent ribosome ‘flavours’ can occur in stressed yeast cells, and is seemingly more pronounced in the translating pool of ribosomes than the non‐translating pool [248]. Stresses, therefore, affect the type of ribosomes and their interaction with tRNAs to favour elongation of specific mRNAs upon stress.

## Effect of stresses on translation termination

Reducing translation termination is a possible further mechanism to increase the abundance of stalled ribosomes and decrease translation. Large‐scale proteomic studies identified no changes in levels of the release factor complex proteins eRF1 and eRF3, or the ribosome recycling protein ABCE1 following UPR induction in mammalian cells [249, 250]. eRF1, eRF3 and ABCE1 remain largely cytosolic following stress, although a fraction of eRF1 and eRF3 have been observed in yeast PBs, and all three are marginally associated with mammalian SGs [251, 252, 253, 254]. Termination factor recruitment to SGs upon oxidative stress is coincident with an increase in stop codon readthrough and free 80S ribosomes, suggestive of a defect in translation termination and subsequent ribosome splitting [142, 254]. Supporting a stress‐induced reduction in ribosome recycling, there is an increase in free, dormant, 80S ribosomes following TORC1 inhibition, potentially mediated by Stm1 in yeast and SREBP1 in mammalian cells [255]. How these changes to termination protein availability affect protein translation needs further investigation through targeted experiments to increase their availability upon stress.

## Future perspectives

Knowledge of the what and how of stress‐induced changes in translation is a critical challenge to our understanding of organismal homeostasis. This broad area, covering alterations in the translation efficiency of an individual mRNA, larger scale proteome realignments and the underlying molecular mechanisms remains a field ripe for discovery. Changes to these processes during the course of ageing and disease will likely have clinical relevance and are an important area for future research. Several kinases are known to play a role in translation, but determining the role that changes in their activity play upon stress to affect translation remains, beyond the well‐characterized ISR, largely unclear. Recent technological advances in RBP identification are likely to aid the identification of further important pathways, while the growing awareness of translational hotspots will add more detail to that currently known. The large spread of technical approaches available will power exciting discoveries for many years to come.

## Conflict of interest

The authors declare no conflict of interest.

## Author contributions

TW: Writing – original draft, reviewing and editing; Figure preparation. AR: Writing – reviewing and editing.
